# Embryonic thermal conditioning and post-hatch heat challenge alter hypothalamic expression of genes related to appetite, thermoregulation, and stress modulation in broiler chicks

**DOI:** 10.3389/fphys.2025.1583958

**Published:** 2025-06-17

**Authors:** Reagan Vaughan, Usman Sulaiman, Annalise Flynn, Fernando Biase, Noam Meiri, Dongmin Liu, Paul Siegel, Mark Cline, Elizabeth Gilbert

**Affiliations:** ^1^ Department of Human Nutrition, Foods, and Exercise, Virginia Polytechnic Institute and State University, Blacksburg, VA, United States; ^2^ School of Animal Sciences, Virginia Polytechnic Institute and State University, Blacksburg, VA, United States; ^3^ Institute of Animal Science, Agricultural Research Organization, Volcani Center, Rishon Leziyyon, Israel; ^4^ School of Neuroscience, Virginia Polytechnic Institute and State University, Blacksburg, VA, United States

**Keywords:** embryonic heat conditioning, heat stress, hypothalamus, transcriptome, broiler chicks

## Abstract

The objective of this study was to determine the effects of an acute heat challenge on day 4 post-hatch on the transcriptome of several brain nuclei associated with thermal regulation, stress, and appetite. These included the paraventricular nucleus (PVN) of the hypothalamus, the pre-optic anterior/hypothalamic area (POAH), and the nucleus of the hippocampal commissure (nCPa), in broilers that were subjected to either control incubation conditions or embryonic heat conditioning (EHC). Nuclei were collected at three timepoints relative to the start of heat challenge (0, 2, and 12 h). Total RNA was isolated, and RNA-sequencing was performed. Transcript abundance was quantified, differentially expressed genes (DEGs) were identified, and Gene Ontology analyses were performed. In the nCPa, 469 DEGs were identified across the three timepoints. There were 0 DEGs at hour 0, 2 at hour 2, and 467 at hour 12. Gene Ontology analysis of nCPa samples at hour 12 revealed enrichment in five biological processes, namely, mitochondrial electron transport, mitochondrial respiratory chain complex 1 assembly, synaptic vesicle lumen acidification, protein export from the nucleus, and aerobic respiration. Most of these genes were downregulated, suggesting reduced activity in these processes in EHC chicks. In the POAH, a total of 18 DEGs were identified, with 0, 18, and 0 at hour 0, 2, and 12, respectively. Fewer differences were observed in the PVN, with only four DEGs identified. All four were upregulated in the EHC group, with two involved in hypothalamic thermal responses: vasoactive intestinal peptide transporter 1 (VIPR1) and caprin family member 2 (CAPRIN2). In the nCPa, no differences were detected between hour 2 and hour 0; however, the comparison between hour 12 and hour 2 yielded 9 DEGs. All except one were downregulated at hour 12. The hour 12 vs. hour 0 comparison revealed 49 DEGs, of which 24 were downregulated at hour 12. The results revealed pathways associated with energy metabolism were altered in response to EHC, with most differences in the nCPa. Surprisingly, the fewest differences were observed in the PVN. The findings highlight potential target regions, such as the nCPa, and metabolic pathways that may help better understand how EHC affects stress responses and energy homeostasis later in life.

## Graphical Abstract

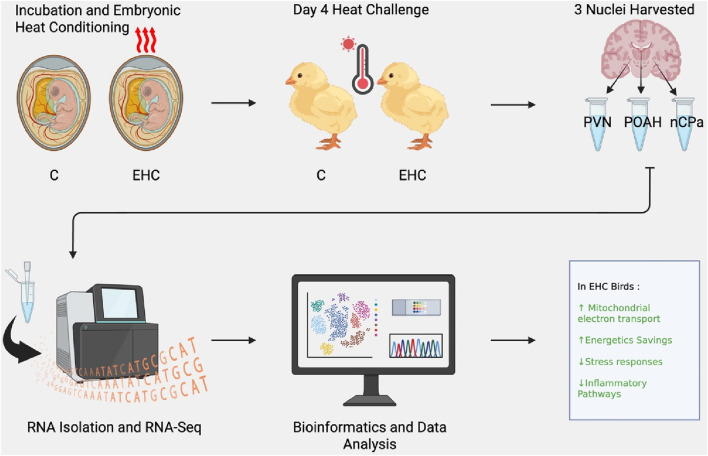

## 1 Introduction

High ambient temperatures are a major environmental factor in the agriculture industry, accounting for annual losses of over $2.36 billion, including $165 million in the poultry industry alone (Nawab et al., 2018). Due to the increases in climate change and the rising global warming crisis, this monetary loss is likely to increase in the coming years, having detrimental effects on the poultry industry and causing a potential food crisis for the growing population. Poultry is the second-highest meat type consumed in the world, following pork. Broiler chicken consumption reached 37.0 million metric tons (mmt) in 1998 and has exponentially increased to 138 mmt by 2022, coinciding with the global population surpassing 8 billion (Dawson, 2023).

Broiler chickens are vulnerable to heat stress due to their selection for high body weight over time. Selection programs have pushed to increase growth, feed-to-gain ratio, and breast muscle size (Reed et al., 2022). Because of these high growth rates, insulation of feathers, and the lack of sweat glands in poultry, heat stress is particularly detrimental in broilers (Rosenberg et al., 2020). These changes have decreased their tolerance to heat, and the impacts of heat stress on broilers account for many physiological issues associated with an increase in the stress response, weight loss, and decreased food intake. These changes often lead to a decrease in meat/carcass production and have resulted in mortalities because of prolonged heat exposure.

In previous studies, we demonstrated that embryonic heat conditioning (EHC) makes chicks more stress-resilient later in life and is associated with improved growth performance, but we have only started to elucidate the underlying molecular mechanisms (Rosenberg et al, 2020; 2022). The use of EHC has shown that animals exposed to thermal stress during the critical part of development display resistance to high temperatures (Labunskay and Meiri, 2006). The hypothalamus is implicated in these effects due to its role in maintaining homeostasis. It integrates central and peripheral signals to modulate endocrine and autonomic output related to the regulation of temperature, appetite, thirst, and other survival-related functions. However, there is little understanding of which hypothalamic nuclei are responsible for the adaptations associated with EHC.

Aside from the agricultural applications, chicks are an ideal model for EHC due to the embryonic environment post-oviposition, allowing for the manipulation of the embryo’s environment. Specific hypothalamic regions of interest are the paraventricular nucleus (PVN), hippocampal commissure (nCPa), and pre-optic anterior hypothalamic area (POAH). Thermoregulation in mammals and avian species (Rothhaas and Chung, 2021) is mediated by the POAH, which is responsible for the innate temperature response and adjusting energy expenditure based on external factors. Heat initiates the stress response, and the POAH projects onto other hypothalamic nuclei associated with stress response, such as the PVN and the dorsomedial hypothalamus (Rothhaas and Chung, 2021; Bohler et al., 2021). The POAH is also responsible for monitoring and integrating temperature alterations from the periphery to maintain homeostasis (Tan and Knight, 2018; Kisliouk et al., 2024). Heat also has intrinsic effects on the response to the stressor in chicks. The initiation of the stress response is through the expression of the corticotropin-releasing factor or hormone (CRF or CRH, respectively). CRF regulates the hypothalamic–pituitary–adrenal (HPA) axis during the stress response and is a potent anorexigenic factor. The nCPa and PVN are both integral in the expression of CRH and for mediating responses to the stressor. The nCPa is essential for the stress response by containing CRH neurons and expressing CRF prior to the PVN, which is understood to maintain and modulate stress in the HPA axis (Kadhim et al., 2019). The PVN, in conjunction with mediating the stressor response, is also essential for modulating feeding behavior and appetite regulation. The PVN regulates water intake and controls food intake by receiving signals from other brain nuclei, with emphasis on neuropeptide Y (NPY) receptors (orexigenic) and melanocortin ligands (anorexigenic) such as pro-opiomelanocortin (POMC) and alpha-melanocyte stimulating hormone (a-MSH) (Yousefvand and Hamidi, 2019). The PVN is intrinsically an anorexigenic center and promotes decreases in appetite due to its population of melanocortin receptors and CRF neurons. Ghrelin stimulates CRF neurons located on the PVN, resulting in decreased food intake (Yousefvand and Hamidi, 2019).

The present study was designed to employ nucleus punch biopsy isolation and RNA sequencing to identify differentially expressed genes and their associated pathways in the nCPa, POAH, and PVN in the control and EHC incubation groups. To help elucidate the effects of EHC on stress resilience, we collected samples from chicks exposed to a heat stress paradigm on day 4 post-hatch. We hypothesized that the identified genes would be related to metabolic and thermoregulatory pathways.

## 2 Materials and methods

### 2.1 Animals

All experimental protocols were approved by the Institutional Animal Care and Use Committee (IACUC) at Virginia Tech. Hubbard x Cobb-500 eggs (*Gallus gallus)* were obtained from a nearby commercial hatchery (same source and flock breeder age as in our previous studies (Sulaiman et al., 2024; Beck et al., 2024)). Upon arrival, eggs were kept at 26.6°C for 12 h and were then randomly divided into the control and EHC groups. These groups were placed in two separate incubators (Rite Farm Products Pro-1056) and labeled as control and EHC, respectively. Both groups from embryonic day (ED) 0–7 were incubated at 37.5°C and 80% relative humidity. The eggs in the control group continued to be incubated at 37.5°C and 80% relative humidity until hatching on ED 21. The EHC group was separated from the control group on ED 7 and introduced to an increased temperature of 39.5°C and 80% relative humidity for 12 h per day (07:30–19:30) and 37.5°C for the remainder of the 24 h until ED 16. The EHC group was then maintained at 37.5°C for the remaining 5 days until hatching. On ED 18.5, all eggs were subjected to candling. Infertile eggs and dead embryos were removed from the study. Eggs with viable embryos were transferred to a common hatching incubator (Rite Farm Products Pro-264) and maintained within their separate groups (separate enclosed trays to prevent mixing of treatments) at 36.9°C and 50% relative humidity for 18 h. The temperature was gradually decreased to 35°C until the hatch was collected. Chicks were arbitrarily divided into group cages based on treatment in preparation for the acute day 4 post-hatch heat challenge. The relative room temperature was 30°C, with *ad libitum* access to feed and water and 24 h continuous light exposure, similar to our previous studies (Sulaiman et al., 2024; Beck et al., 2024).

### 2.2 Acute heat challenge

On day 4 post-hatch, control and EHC groups were subjected to an acute heat challenge at 36°C. The temperature of 36°C was carefully selected based on preliminary trials designed to induce observable behavioral indicators of heat stress, such as panting and wing spreading while avoiding more severe physiological responses. Punch samples were collected at three timepoints: 0 h (before heat challenge; baseline), 2 h, and 12 h from the start of the increased temperature. Within these three timepoints, 10 chicks were randomly collected and euthanized from the control and EHC groups (20 total) for each timepoint. Chicks were individually weighed, euthanized by decapitation, and sexed *via* gonad identification.

### 2.3 Punch biopsy collection

Three hypothalamic nuclei, namely, the PVN, POAH, and nCPa, were collected from each bird (n = 60), yielding a total of 180 samples. Brains were perfused with 1.5 mL of RNA stabilizing solution *via* the carotid artery based on methods used by Bohler et al. (2020). Immediately after perfusion, brains were processed into tissue blocks, mounted on chucks with OCT embedding medium, and sectioned at −15°C in a cryostat. Brain sections were cut at a thickness of 500 µm, and biopsies were collected based on anatomical landmarks described in the stereotaxic atlas by Kuenzel and Masson (1988). The nCPa was the most rostral, referring to 8.2 mm in the stereotaxic atlas. The POAH was next at 7.8 mm, and the PVN was collected at 6.8 mm. Punches were collected using disposable 1-mm biopsy punch instruments (Integra Lifesciences Corp, Princeton, NJ). Anatomy and biopsy collection were confirmed using a digitized stereotaxic atlas, with photos of tissue sections overlaid on the atlas; the methods were further described by Bohler et al. (2020). After collection, the punches were immediately submerged in 1.5 mL microcentrifuge tubes containing RNA lysis buffer (Norgen Biotek, Thorold, ON, Canada) and 1% beta-mercaptoethanol (Calbiochem, San Diego, CA, United States). The tubes were vortexed, snap-frozen in liquid nitrogen, and stored at −80°C until RNA isolation was performed.

### 2.4 Total RNA extraction, cDNA synthesis, and real-time quantitative PCR

Total RNA was isolated using the Total RNA Purification Micro Kit (Norgen Biotek, Thorold, ON, Canada), according to the manufacturer’s instructions. Samples were thawed to room temperature (3 min) and vortexed for 30 s. An amount of 100 μL of 70% molecular-biology-grade ethanol was added to the lysate, and then steps were performed according to the kit’s instructions. Purity and concentration of the total RNA were analyzed at 260/280/230 ng/μL using the NanoPhotometer Pearl Spectrophotometer (Implen, Westlake Village, CA, United States), according to the manufacturer’s instructions. The High-Capacity cDNA Reverse Transcription Kit (Applied Biosystems, Carlsbad, CA, United States) was used to synthesize the first-strand complementary DNA (cDNA) from 100 ng of total RNA in 20 µL reactions, according to the manufacturer’s protocol. Reactions were performed under the following conditions: 25°C for 10 min, 37°C for 120 min, and 85°C for 5 min. Primers (Table 1) for RT-qPCR were designed using Primer Express software (Applied Biosystems, Carlsbad, CA, United States), following the manufacturer’s instructions. RT-PCRs were performed in duplicate using 10 µL reaction volumes, each containing 5 µL of Fast SYBR Green Master Mix (Applied Biosystems, Carlsbad, CA, United States). PCR was performed as follows: at 95°C for 20 s, followed by 40 cycles at 90°C for 3 s, and at 60°C for 30 s. A dissociation step at 95°C for 15 s, 60°C for 1 min, 95°C for 15 s, and 60°C for 15 s was performed at the end of each PCR to ensure amplicon specificity. PCR data were analyzed using JMP Pro 16 (SAS Institute Inc., Cary, NC) with the ΔΔCT method, with β-actin as the reference gene, and the calibrator sample of each nucleus was the average of the control chicks per group. The statistical model included the effect of treatment (EHC vs control), timepoints (0, 2, and 12 h), and the interactions between them.

**TABLE 1:** Primers used for real-time PCR.

Gene	Sequences (forward/reverse)	Accession no.
β-Actin	GTCCACCGCAAATGCTTCTAA/TGCGCATTTATGGGTTTTGTT	NM_205,518.2
NPY	CATGCAGGGCACCATGAG/CAGCGACAAGGCGAAAGTC	NM_205,473.2
CRH	TCAGCACCAGAGCCATCACA/GCTCTATAAAAATAAAGAGGTGACATCAGA	NM_001123031.1
POMC	GCCAGACCCCGCTGATG/CTTGTAGGCGCTTTTGACGAT	NM_001398117.1
CCK	GGAAGGAAGGGAAGGAGGAA/GAGGAGCACGCAGATGCA	NM_001001741.2
UCN3	GGGCCTTCCGTCTCTACAATG/GGTGAGGGCCGTGTTGAG	XM_001231710.6
TRH	GCAGAAAATCACAATGCCATCTAT/CACCAGACAAGGTCAGGCAAA	NM_001030383.2
HSP90	GCAGCAGCTGAAGGAATTTGA/GGAAGCTCTAAGCCCTCTTTTGT	NM_001109785.2

Abbreviations: NPY, neuropeptide Y; CRF, corticotropin-releasing factor; POMC, proopiomelanocortin; CCK, cholecystokinin; UCN3, urocortin-3; TRH, thyrotropin-releasing hormone; HSP90, heat shock protein 90.

### 2.5 Selection of chick samples for RNA-sequencing and production of sequencing data

Selection of samples was based on concentrations (ng/µL) and the ability to use the three nuclei samples from the same bird. A total of 72 samples were submitted to the Genomic Sequencing Center at Virginia Tech (Blacksburg, VA, United States). The 72 samples were collected from 24 total chicks (three samples per chick, n = 8 chicks per timepoint). All the 72 samples were split to account for 36 EHC and 36 control samples. Among the selected chicks, 12 were male and 12 were female; however, sex did not prove to be significantly different. NanoDrop concentrations from the Pearl Spectrophotometer ranged from 4.8 ng/μL to 29.2 ng/μL with an average of 13.5 ng/μL. Each sample submitted was strictly between 16 and 18 µL. The integrity of RNA was verified using High Sensitivity RNA ScreenTape Assays for TapeStation (Agilent).

### 2.6 RNA-seq library preparation

RNA samples were converted into a strand-specific library using Illumina Stranded Total RNA Prep, Ligation with Ribo-Zero Plus Sample Prep Kit (Illumina, 20040529) for subsequent cluster generation and sequencing on Illumina’s NovaSeq 6000. The libraries were enriched by 13 cycles of PCR, quality-checked using Agilent TapeStation, and quantitated by qPCR. Individually indexed cDNA libraries were pooled and sequenced on a NovaSeq 6000 S2 (200 cycle, PE) using Illumina NovaSeq Control Software (version 1.8.0.). Binary base call (BCL) files were converted to FASTQ files, and adapters were trimmed and demultiplexed using bcl2fastq Conversion Software. The FASTQ files were provided by the Genomic Sequencing Center for further analysis.

### 2.7 RNA-seq alignment, processing of sequences, and gene quantification

First, adapters were removed from the sequences using Trimmomatic (v 0.39), retaining only those with a length of ≥100 nucleotides and an average quality score ≥30. We aligned the reads to the chicken genome (galgal1) using HISAT2 (v 2.2.1) (Kim et al., 2019). Next, we used SAMtools (v 1.10) (Li et al., 2009) to retain the reads with one match to the genome, followed by removing duplicates using bammarkduplicates from biobambam2 (v 2.0.95) (Tischler and Leonard, 2014). Samples with 90% or greater alignment accuracy were used for counting.

featureCounts (v 2.0.1) (Liao et al., 2014) was used to count reads according to the ensemble annotation (Gallus_gallus.bGalGal1.mat.broiler.GRCg7b.110.gtf). We quantified counts per million (CPM) and fragments per kilobase per million (FPKM) using the functions “cpm” or “rpkm” from the “edgeR” package (McCarthy et al., 2012; Robinson et al., 2010). We also calculated transcript per million using the formula presented by Li and Dewey (2011). We retained genes annotated as long non-coding RNA (lncRNA), protein-coding, and pseudogenes (Robinson et al., 2010). Genes were only used for further analysis if the CPM and FPKM were >1 in at least four samples. This approach was adopted to reduce the number of genes with low expression and, thus, prone to producing confounding results (Bourgon et al., 2010).

### 2.8 Analysis of differential transcript abundance

Transcript abundance between samples from each group was compared using the R packages “edgeR” (McCarthy et al., 2012; McCarthy and Smyth, 2012), with the quasi-likelihood test and “DEseq2” (Love et al., 2014), using the Wald and likelihood tests. We also added sex as a fixed effect in the model. The nominal *p-*values of both tests were corrected for multiple hypothesis testing using the false discovery rate (FDR) method (Benjamini and Hochberg, 1995). Differential transcript abundance was assumed when FDR ≤0.1 for both tests and the |Log fold change (logFC)| > 1.

### 2.9 Enrichment of Gene Ontology

Tests for enrichment of Gene Ontology (GO) categories were carried out using the “goseq” package (Young et al., 2010) in R software. In all tests, we used the genes whose transcript abundances were estimated for the samples being tested as the background. The nominal *p*-value was adjusted for multiple hypothesis testing by controlling the familywise error rate following the method proposed by Holm (1979) using the function “p.adjust” from the “stats” R package. Significance was acknowledged when FWER ≤0.1.

## 3 Results

### 3.1 RNA-seq analysis

**FIGURE 1:**
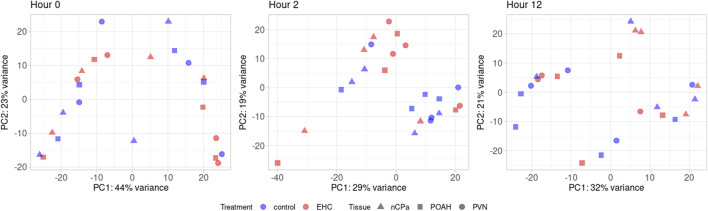
Principal component analysis using 17,584 quantified genes. Among these are the nCPa (n = 4/treatment/timepoint), POAH (n = 4/treatment/timepoint), and PVN (n = 4/treatment/timepoint) tissues from EHC (n = 12/timepoint) and control (n = 12/timepoint).

From RNA sequencing, a total of 25,067,562 fragment sequence reads were obtained, and an average of 4,217,491 pairs of reads per sample were mapped to the chicken genome (Hillier et al., 2004). There were 17,584 total genes identified, with 3,403 being lncRNA, 14,160 being protein-coding, and 21 being pseudogenes. Among these genes, there were a total of 491 DEGs across the nCPa, POAH, and PVN when comparing EHC vs control (C) at different timepoints. The principal component analysis (Figure 1) visualizes our dataset separated by timepoints into three graphs. Within these graphs, EHC samples are represented in red and control is represented in blue. The nuclei are differentiated by shape, with POAH as squares, PVN as circles, and nCPa as triangles. There was no explicit separation of chicks based on treatment or tissue when all 17,584 genes were included for a principal component analysis.

### 3.2 Differential gene expression in EHC chicks relative to control

#### 3.2.1 DEGs identified in the nCPa

For the three different nuclei assessed, there was variation in the numbers and types of differentially expressed genes (DEGs) for EHC chicks vs. control at the different timepoints in the heat challenge. In the nCPa, there were a total of 469 DEGs identified across all three timepoints. There were 0 DEGs identified at hour 0. At hour 2, in the nCPa, there were 2 DEGs, namely, keratin 13 and phosphoserine aminotransferase 1, both of which were downregulated in the EHC group (Table 2). With only 2 DEGs in hour 2 in nCPa EHC chicks, we investigated the functions of these genes individually rather than performing an enrichment analysis.

**TABLE 2:** DEGs in EHC chicks at hour 2 in nCPa nuclei^a^.

Direction	Ensembl id	Name	Description	logFC	P-value	FDR
Downregulated	ENSGALG00010022872	KRT13	Keratin 13	−4.43240	2.14E-06	0.03771
Downregulated	ENSGALG00010015533	PSAT1	Phosphoserine aminotransferase 1	−0.96427	1.06E-05	0.09292

Abbreviations: KRT13, keratin 13; PSAT1, phosphoserine aminotransferase 1.

^a^ All results were determined with FDR <0.1.

Hour 12 nCPa tissues yielded a total of 467 DEGs. We selected the 10 highest upregulated and 10 lowest downregulated genes for tabular display (Table 3). Because of the large number of DEGs, we performed an enrichment of GO test on the hour 12 nCPa sample data. On completing the GO enrichment, 24 genes were considered significantly enriched with a FWER ≤0.1. Among these, five biological processes were enriched, namely, mitochondrial electron transport, mitochondrial respiratory chain complex 1 assembly, synaptic vesicle lumen acidification, protein export from nucleus, and aerobic respiration (Table 4). Twenty one out of the 24 genes were downregulated, suggesting that these biological processes were less functional/depleted in EHC chicks.

**TABLE 3:** Ten most upregulated and 10 most downregulated DEGs in EHC at hour 12 in nCPa tissue^a^.

Direction	Ensembl ID	Name	Description	logFC	P-value	FDR
Upregulated	ENSGALG00010000384			0.97207	1.76E-08	5.72E-05
Upregulated	ENSGALG00010005981	AREL1	Apoptosis-resistant E3 ubiquitin protein ligase 1	0.81544	4.16E-07	5.62E-04
Upregulated	ENSGALG00010023442	STK25	Serine/threonine kinase 25	0.42320	7.56E-06	0.00415
Upregulated	ENSGALG00010017973	PKD1	Polycystin 1, transient receptor potential channel interacting	0.70729	1.46E-05	0.00677,265
Upregulated	ENSGALG00010016544	ADCY9	Adenylate cyclase 9	0.47785	2.03E-05	0.00791,217
Upregulated	ENSGALG00010020608	CAPZB	Capping actin protein of muscle Z-line beta subunit	0.52601	2.17E-05	0.00795,085
Upregulated	ENSGALG00010013908	TBC1D20	TBC1 domain family member 20	0.46503	3.07E-05	0.00967,321
Upregulated	ENSGALG00010018022	XPO6	Exportin 6	0.46862	3.20E-05	0.00971,448
Upregulated	ENSGALG00010026900			0.68841	3.67E-05	0.01046038
Upregulated	ENSGALG00010028151	DOCK3	Dedicator of cytokinesis 3	0.71350	3.67E-05	0.01046038
Downregulated	ENSGALG00010008407	GNAI1	G protein subunit alpha i1	−1.38534	1.57E-10	2.05E-06
Downregulated	ENSGALG00010023910	RNF7	Ring finger protein 7	−1.15010	2.33E-10	2.05E-06
Downregulated	ENSGALG00010007903	PSMA2	Proteasome subunit alpha 2	−1.09620	8.55E-09	5.01E-05
Downregulated	ENSGALG00010021639	SCG5	Secretogranin V	−1.91617	1.44E-08	5.72E-05
Downregulated	ENSGALG00010016240	EIF4A2	Eukaryotic translation initiation factor 4A2	−0.66176	1.95E-08	5.72E-05
Downregulated	ENSGALG00010014967	HNRNPH1	Heterogeneous nuclear ribonucleoprotein H1	−1.01498	4.32E-08	1.08E-04
Downregulated	ENSGALG00010024777			−2.48793	5.93E-08	1.30E-04
Downregulated	ENSGALG00010001738	PUDP	Pseudouridine 5′-phosphatase	−1.00633	1.34E-07	2.60E-04
Downregulated	ENSGALG00010000011	ND2	NADH dehydrogenase subunit 2	−3.19799	1.52E-07	2.60E-04
Downregulated	ENSGALG00010000022	ATP8	ATP synthase F0 subunit 8	−2.74138	1.63E-07	2.60E-04

Apoptosis-resistant E3 ubiquitin protein ligase 1 (AREL1), serine/threonine kinase 25 (STK25), polycystin 1, transient receptor potential channel interacting (PKD1), adenylate cyclase 9 (ADCY9), capping actin protein of muscle Z-line beta subunit (CAPZB), TBC1 domain family member 20 (TBC1D20), exportin 6 (XPO6), dedicator of cytokinesis 3 (DOCK3), G protein subunit alpha i1 (GNAI1), ring finger protein 7 (RNF7), proteasome subunit alpha 2 (PSMA2), secretogranin V (SCG5), eukaryotic translation initiation factor 4A2 (EIF4A2), heterogeneous nuclear ribonucleoprotein H1 (HNRNPH1), pseudouridine 5′-phosphatase (PUDP), NADH dehydrogenase subunit 2 (ND2), and ATP synthase F0 subunit 8 (ATP8). Abbreviations are not provided for unannotated genes.

^a^ All DE results were determined with FDR <0.1.

**TABLE 4:** DEGs after Gene Ontology enrichment and biological process category; FWER ≤0.1.

Ensembl gene ID	Gene name	Overrepresented p-value	Number DE in category	Total number in category	Biological process	FWER	Fold enrichment	Fold change
ENSGALG00010000011	ND2	7.03E-10	10	23	Mitochondrial electron transport, NADH to ubiquitone	6.12E-08	14.90296	−3.19799
ENSGALG00010000029	ND4	7.03E-10	10	23	Mitochondrial electron transport, NADH to ubiquitone	6.12E-08	14.90296	−2.84102
ENSGALG00010000037	ND6	7.03E-10	10	23	Mitochondrial electron transport, NADH to ubiquitone	6.12E-08	14.90296	−2.17901
ENSGALG00010000007	ND1	7.03E-10	10	23	Mitochondrial electron transport, NADH to ubiquitone	6.12E-08	14.90296	−2.01385
ENSGALG00010000033	ND5	7.03E-10	10	23	Mitochondrial electron transport, NADH to ubiquitone	6.12E-08	14.90296	−1.75243
ENSGALG00010003358	DNAJC15	7.03E-10	10	23	Mitochondrial electron transport, NADH to ubiquitone	6.12E-08	14.90296	−1.35734
ENSGALG00010000026	ND3	7.03E-10	10	23	Mitochondrial electron transport, NADH to ubiquitone	6.12E-08	14.90296	−1.34728
ENSGALG00010008731	NDUFB9	7.03E-10	10	23	Mitochondrial electron transport, NADH to ubiquitone	6.12E-08	14.90296	−1.12493
ENSGALG00010028036	NDUFS7	7.03E-10	10	23	Mitochondrial electron transport, NADH to ubiquitone	6.12E-08	14.90296	−1.12090
ENSGALG00010007555	NDUFS6	7.03E-10	10	23	Mitochondrial electron transport, NADH to ubiquitone	6.12E-08	14.90296	−1.04794
ENSGALG00010000011	ND2	1.70E-07	11	43	Mitochondrial respiratory chain complex I assembly	1.46E-05	8.76848	−3.19799
ENSGALG00010000029	ND4	1.70E-07	11	43	Mitochondrial respiratory chain complex I assembly	1.46E-05	8.76848	−2.84102
ENSGALG00010000037	ND6	1.70E-07	11	43	Mitochondrial respiratory chain complex I assembly	1.46E-05	8.76848	−2.17901
ENSGALG00010000007	ND1	1.70E-07	11	43	Mitochondrial respiratory chain complex I assembly	1.46E-05	8.76848	−2.01385
ENSGALG00010000033	ND5	1.70E-07	11	43	Mitochondrial respiratory chain complex I assembly	1.46E-05	8.76848	−1.75243
ENSGALG00010018137	NDUFB5	1.70E-07	11	43	Mitochondrial respiratory chain complex I assembly	1.46E-05	8.76848	−1.35734
ENSGALG00010017738	NDUFS5	1.70E-07	11	43	Mitochondrial respiratory chain complex I assembly	1.46E-05	8.76848	−1.34728
ENSGALG00010008731	NDUFB9	1.70E-07	11	43	Mitochondrial respiratory chain complex I assembly	1.46E-05	8.76848	−1.12493
ENSGALG00010028036	NDUFS7	1.70E-07	11	43	Mitochondrial respiratory chain complex I assembly	1.46E-05	8.76848	−1.12090
ENSGALG00010004123	NDUFAF4	1.70E-07	11	43	Mitochondrial respiratory chain complex I assembly	1.46E-05	8.76848	−0.61710
ENSGALG00010024338	NDUFA9	1.70E-07	11	43	Mitochondrial respiratory chain complex I assembly	1.46E-05	8.76848	−0.37826
ENSGALG00010010906	ATP6V1C1	1.85E-04	4	8	Synaptic vesicle lumen acidification	0.01570	17.13840	−1.19382
ENSGALG00010004370	ATP6V1H	1.85E-04	4	8	Synaptic vesicle lumen acidification	0.01570	17.13840	−0.86649
ENSGALG00010028896	ATP6V1G1	1.85E-04	4	8	Synaptic vesicle lumen acidification	0.01570	17.13840	−0.72372
ENSGALG00010020015	ATP6V1D	1.85E-04	4	8	Synaptic vesicle lumen acidification	0.01570	17.13840	−0.60052
ENSGALG00010010262	HSPA9	9.57E-04	5	22	Protein export from nucleus	0.08035	7.79018	−0.53047
ENSGALG00010028137	NUP214	9.57E-04	5	22	Protein export from nucleus	0.08035	7.79018	0.46482
ENSGALG00010018022	XPO6	9.57E-04	5	22	Protein export from nucleus	0.08035	7.79018	0.46862
ENSGALG00010020305	XPO7	9.57E-04	5	22	Protein export from nucleus	0.08035	7.79018	0.49686
ENSGALG00010017973	PKD1	9.57E-04	5	22	Protein export from nucleus	0.08035	7.79018	0.70729
ENSGALG00010000029	ND4	1.03E-03	4	14	Aerobic respiration	0.08582	9.79337	−2.84102
ENSGALG00010000007	ND1	1.03E-03	4	14	Aerobic respiration	0.08582	9.79337	−2.01385
ENSGALG00010000017	COX1	1.03E-03	4	14	Aerobic respiration	0.08582	9.79337	−1.27152
ENSGALG00010022937	BLOC1S1	1.03E-03	4	14	Aerobic respiration	0.08582	9.79337	−1.1705

#### 3.2.2 DEGs identified in the POAH

In the POAH, a total of 18 DEGs were identified after performing edgeR and DEseq2 tests. Similar to the nCPa, at hour 0, there were 0 DEGs identified between treatment groups. At hour 2, there were 18 DEGs (Table 5). These genes were investigated individually as there were not enough genes to perform a robust enrichment analysis. All but one gene were upregulated (BEND3), 17 genes were protein-coding, and 1(ENSGALG00010004520) was lncRNA. GO enrichment was not performed. At hour 12, no DEGs were identified.

**TABLE 5:** Differentially expressed genes in the EHC group vs control group at hour 2 in the POAH^a^.

Direction	Ensembl ID	Name	Description	logFC	P-value	FDR
Upregulated	ENSGALG00010004520			1.85130	1.28E-05	0.06251
Upregulated	ENSGALG00010003429			1.94931	1.34E-05	0.06251
Upregulated	ENSGALG00010017487	C1QTNF8	C1q and TNF-related 8	5.44959	1.78E-05	0.06251
Upregulated	ENSGALG00010003395			1.86689	1.80E-05	0.06251
Upregulated	ENSGALG00010029651	CHAD		5.30853	3.09E-05	0.06251
Upregulated	ENSGALG00010003346			1.75279	3.33E-05	0.06251
Upregulated	ENSGALG00010022764	MFAP5	Microfibril-associated protein 5	2.54772	3.50E-05	0.06251
Upregulated	ENSGALG00010003368			1.87428	3.59E-05	0.06251
Upregulated	ENSGALG00010011181			1.37643	3.62E-05	0.06251
Upregulated	ENSGALG00010003377			1.78224	3.80E-05	0.06251
Upregulated	ENSGALG00010012092	OC3	Osteocalcin-like protein OC3	6.42273	3.91E-05	0.06251
Upregulated	ENSGALG00010003408			1.74615	5.20E-05	0.07613
Upregulated	ENSGALG00010029387	SERPINF1	Serpin family F member 1	3.53737	8.04E-05	0.09889
Upregulated	ENSGALG00010012083	MGP	Matrix Gla protein	6.29646	8.60E-05	0.09889
Downregulated	ENSGALG00010012529	BEND3	BEN domain containing 3	−0.67962	8.73E-05	0.09889
Upregulated	ENSGALG00010029270	TIMP2	TIMP metallopeptidase inhibitor 2	1.01350	9.49E-05	0.09889
Upregulated	ENSGALG00010003202	FAM46A	Family with sequence similarity 46 member A	1.57178	9.70E-05	0.09889
Upregulated	ENSGALG00010000651			4.04485	1.01E-04	0.09889

Microfibril-associated protein 5 (MFAP5), osteocalcin-like protein OC3 (OC3), serpin family F member 1 (SERPINF1), matrix Gla protein (MGP), BEN domain containing 3 (BEND3), TIMP metallopeptidase inhibitor 2 (TIMP2), and family with sequence similarity 46 member A (FAM46A). Abbreviations are not provided for unannotated genes.

^a^ All DE results were determined with FDR <0.1. C1q and TNF-related 8 (C1QTNF8).

#### 3.2.3 DEGs identified in the PVN

Fewer differences were observed in the PVN than in the nCPa and POAH, with only four DEGs identified (Table 6). Four of these were different at hour 2. At hour 0 and 12, there were no DEGs. The four genes that were differentially expressed were upregulated in the EHC group. Among these four, two are associated with hypothalamic responses: vasoactive intestinal peptide receptor 1 (VIPR1) and caprin family member 2 (CAPRIN2) Bárez-López et al., 2022). Of the other two, one gene encodes a factor involved in mitochondrial calcium uptake (MICU1) and the other encodes an actin-binding protein, Kaptin (KPTN).

**TABLE 6:** Differentially expressed genes in EHC vs control chicks at hour 2 in the paraventricular nucleus^a^.

Direction	Ensembl ID	Name	Description	logFC	P-value	FDR
Upregulated	ENSGALG00010027363	VIPR1	Vasoactive intestinal peptide receptor 1	1.64135	3.77E-06	0.06319
Upregulated	ENSGALG00010010774	KPTN	Kaptin, actin-binding protein	2.40213	1.01E-05	0.06319
Upregulated	ENSGALG00010021481	MICU1	Mitochondrial calcium uptake 1	0.84260	1.08E-05	0.06319
Upregulated	ENSGALG00010012072	CAPRIN2	Caprin family member 2	1.31568	2.16E-05	0.09515

Vasoactive intestinal peptide receptor 1 (VIPR1), Kaptin, actin binding protein (KPTN), mitochondrial calcium uptake 1 (MICU1), and caprin family member 2 (CAPRIN2).

^a^ All DE results were determined with FDR <0.1.

### 3.3 Differential gene expression relative to 0-, 2-, and 12-h timepoints irrespective of EHC vs control

The differences described in Section 3.2 were detected by comparing the two treatment groups, EHC and control. We also completed analyses that determined the effect of timepoints in the heat challenge, irrespective of treatment. Timepoint comparisons were made as follows: hour 12 relative to hour 2 (12 vs 2), hour 2 vs hour 0, and hour 12 vs hour 0 within tissue. There were no DEGs identified in the POAH or PVN.

No differences were detected in the nCPa for hour 2 vs hour 0; however, the comparison between hour 12 and hour 2 yielded 9 DEGs (Table 7). All genes, except for ubiquitin-associated protein 2-like (UBAP2L), were downregulated at hour 12 relative to hour 2.

**TABLE 7:** DEGs identified at hour 12 relative to those identified at hour 2 in the nCPa^a^.

Direction	Ensembl ID	Name	Description	logFC	P-value	FDR
Downregulated	ENSGALG00010014243	HIGD2A		−1.32130	4.20E-06	0.05620
Downregulated	ENSGALG00010016502	YIPF5	Yip1 domain family member 5	−0.60054	6.39E-06	0.05620
Downregulated	ENSGALG00010019810	ZFAND6	Zinc finger AN1-type containing 6	−0.68436	1.28E-05	0.05747
Upregulated	ENSGALG00010027867	UBAP2L	Ubiquitin-associated protein 2 like	0.56530	1.31E-05	0.05747
Downregulated	ENSGALG00010011991	CSNK1A1	Casein kinase 1 alpha 1	−0.43456	1.79E-05	0.06285
Downregulated	ENSGALG00010027359			−1.03567	3.02E-05	0.08838
Downregulated	ENSGALG00010028459	MCL1	BCL2 family apoptosis regulator	−0.50885	4.68E-05	0.09720
Downregulated	ENSGALG00010027617			−0.43455	4.72E-05	0.09720
Downregulated	ENSGALG00010028823	GFI1B	Growth factor independent 1B transcriptional repressor	−3.11826	4.98E-05	0.09720

Yip1 domain family member 5 (YIPF5), zinc finger AN1-type containing 6 (ZFAND6), ubiquitin-associated protein 2 like (UBAP2L), casein kinase 1 alpha 1 (CSNK1A1), BCL2 family apoptosis regulator (MCL1), and growth factor independent 1B transcriptional repressor (GFI18). Abbreviations are not provided for unannotated genes.

^a^ All DE results were determined with FDR <0.1.

In the nCPa, the hour 12 vs hour 0 comparison revealed 49 DEGs (Table 8). Of these genes, 16 were upregulated and 24 genes were downregulated at hour 12. After individually investigating each DEG, it was observed that these genes were classified as protein-coding or lncRNA and that to our knowledge, no existing literature links these genes to either hypothalamic regulation or control.

**TABLE 8:** DEGs at hour 12 vs hour 0 in the nCP^a^.

Direction	Ensembl ID	Name	Description	logFC	P-value	FDR
Downregulated	ENSGALG00010024651	PSMD3	Proteasome 26S subunit, non-ATPase 3	−0.68594	2.15E-06	0.02871
Downregulated	ENSGALG00010012032			−1.28093	3.27E-06	0.02871
Downregulated	ENSGALG00010014243	HIGD2A		−1.27669	5.64E-06	0.03307
Downregulated	ENSGALG00010006120	MOGS	Mannosyl-oligosaccharide glucosidase	−1.00933	8.47E-06	0.03723
Downregulated	ENSGALG00010011531	MTDH	Metadherin	−0.44060	1.52E-05	0.05334
Downregulated	ENSGALG00010020476	TMEM132A	Transmembrane protein 132A	−0.53498	1.98E-05	0.05796
Downregulated	ENSGALG00010021416	PLEKHH3	Pleckstrin homology, MyTH4, and FERM domain containing H3	−0.78872	3.03E-05	0.06675
Downregulated	ENSGALG00010028008	NTMT1	N-terminal Xaa-Pro-Lys N-methyltransferase 1	−0.53559	3.89E-05	0.06675
Downregulated	ENSGALG00010006065	WDR54	WD repeat domain 54	−0.68781	5.49E-05	0.06675
Upregulated	ENSGALG00010024961	LSM12	LSM12 homolog	0.66363	6.03E-05	0.06675
Downregulated	ENSGALG00010019064	DPY19L1		−0.60439	6.30E-05	0.06675
Upregulated	ENSGALG00010010126			1.77888	6.44E-05	0.06675
Downregulated	ENSGALG00010014278	G3BP1	G3BP stress granule assembly factor 1	−0.48920	6.73E-05	0.06675
Downregulated	ENSGALG00010008172	SNTB2	Syntrophin beta 2	−1.04640	7.09E-05	0.06675
Downregulated	ENSGALG00010015299	GALE	UDP-galactose-4-epimerase	−0.64200	7.17E-05	0.06675
Downregulated	ENSGALG00010024635			−0.86725	7.43E-05	0.06675
Downregulated	ENSGALG00010027617			−0.41535	7.81E-05	0.06675
Downregulated	ENSGALG00010025323	KLHDC8A	Kelch domain containing 8A	−0.48698	8.10E-05	0.06675
Downregulated	ENSGALG00010002792			−0.64896	8.28E-05	0.06675
Upregulated	ENSGALG00010001837	TAB3	TGF-beta activated kinase 1 (MAP3K7) binding protein 3	0.34290	8.42E-05	0.06675
Downregulated	ENSGALG00010018482	C10orf2	Twinkle mtDNA helicase	−0.89467	8.67E-05	0.06675
Upregulated	ENSGALG00010002185			0.94745	9.03E-05	0.06675
Downregulated	ENSGALG00010018951	YKT6	YKT6 v-SNARE homolog (*S. cerevisiae*)	−0.71108	9.11E-05	0.06675
Upregulated	ENSGALG00010001744			0.94319	1.12E-04	0.07224
Upregulated	ENSGALG00010029333			0.59066	1.24E-04	0.07224
Upregulated	ENSGALG00010029624	RASL10B	RAS-like family 10 member B	0.61430	1.33E-04	0.07224
Downregulated	ENSGALG00010022034			−0.69617	1.33E-04	0.07224
Upregulated	ENSGALG00010000022	ATP8	ATP synthase F0 subunit 8	2.64221	1.39E-04	0.07224
Downregulated	ENSGALG00010022513	NDUFAF1	NADH: ubiquinone oxidoreductase complex assembly factor 1	−0.41088	1.40E-04	0.07224
Downregulated	ENSGALG00010027359			−0.90077	1.45E-04	0.07224
Upregulated	ENSGALG00010000011	ND2	NADH dehydrogenase subunit 2	3.16881	1.53E-04	0.07224
Downregulated	ENSGALG00010004991	FAM133B	Family with sequence similarity 133 member B	−0.40436	1.62E-04	0.07224
Downregulated	ENSGALG00010019042	PEX6	Peroxisomal biogenesis factor 6	−0.43292	1.62E-04	0.07224
Downregulated	ENSGALG00010023482	SOGA1	Suppressor of glucose, autophagy associated 1	−0.42100	1.66E-04	0.07224
Upregulated	ENSGALG00010000020	COX2	Cytochrome c oxidase subunit II	1.06082	1.73E-04	0.07224
Upregulated	ENSGALG00010000033	ND5	NADH dehydrogenase subunit 5	1.68731	1.75E-04	0.07224
Upregulated	ENSGALG00010000026	ND3	NADH dehydrogenase subunit 3	1.56277	1.77E-04	0.07224
Downregulated	ENSGALG00010020451			−0.63924	2.09E-04	0.07952
Downregulated	ENSGALG00010020970	C6H10orf76	Armadillo-like helical domain containing 3	−0.46178	2.17E-04	0.07952
Downregulated	ENSGALG00010018044	INTS11	Integrator complex subunit 11	−1.44967	2.22E-04	0.07952
Upregulated	ENSGALG00010000023	ATP6	ATP synthase F0 subunit 6	2.29204	2.29E-04	0.08025
Downregulated	ENSGALG00010008010	CERK	Ceramide kinase	−0.56402	2.36E-04	0.08025
Upregulated	ENSGALG00010000029	ND4	NADH dehydrogenase subunit 4	2.66278	2.37E-04	0.08025
Downregulated	ENSGALG00010027533	WDR18	WD repeat domain 18	−0.76318	2.69E-04	0.08749
Downregulated	ENSGALG00010028129	CYB561D2	Cytochrome b561 family member D2	−0.81944	2.84E-04	0.08908
Downregulated	ENSGALG00010013402	MARCKS	Myristoylated alanine rich protein kinase C substrate	−0.52691	3.04E-04	0.09188
Downregulated	ENSGALG00010029763	GEMIN4	Gem nuclear organelle associated protein 4	−0.50494	3.08E-04	0.09188
Upregulated	ENSGALG00010010111	CNOT4	CCR4-NOT transcription complex subunit 4	0.38662	3.21E-04	0.09254
Upregulated	ENSGALG00010000034	CYTB	Cytochrome b	1.99713	3.27E-04	0.09272

Proteasome 26S subunit, non-ATPase 3 (PSMD3), mannosyl-oligosaccharide glucosidase (MOGS), metadherin (MTDH), transmembrane protein 132A (TMEM132A), pleckstrin homology, MyTH4, and FERM domain containing H3 (PLEKHH3), N-terminal Xaa-Pro-Lys N-methyltransferase 1 (NTMT1), WD repeat domain 54 (WDR54), LSM12 homolog (LSM12), G3BP stress granule assembly factor 1 (G3BP1), syntrophin beta 2 (SNTB2), UDP-galactose-4-epimerase (GALE), kelch domain containing 8A (KLHDC8A), TGF-beta activated kinase 1 (MAP3K7) binding protein 3 (TAB3), twinkle mtDNA helicase (C10orf2), YKT6 v-SNARE homolog (*S. cerevisiae*) (YKT6), RAS like family 10 member B (RASL10B), ATP synthase F0 subunit 8 (ATP8), NADH: ubiquinone oxidoreductase complex assembly factor 1 (NDUFAF1), NADH dehydrogenase subunit 2 (ND2), family with sequence similarity 133 member B (FAM133B), peroxisomal biogenesis factor 6 (PEX6), suppressor of glucose, autophagy associated 1 (SOGA1), cytochrome c oxidase subunit II (COX2), NADH dehydrogenase subunit 5 (ND5), NADH dehydrogenase subunit 3 (ND3), armadillo like helical domain containing 3 (C6H10orf76), integrator complex subunit 11 (INTS11), ATP synthase F0 subunit 6 (ATP6), ceramide kinase (CERK), NADH dehydrogenase subunit 4 (ND4), WD repeat domain 18 (WDR18), cytochrome b561 family member D2 (CYB561D2), myristoylated alanine rich protein kinase C substrate (MARCKS), gem nuclear organelle associated protein 4 (GEMIN4), CCR4-NOT transcription complex subunit 4 (CNOT4), and cytochrome b (CYTB). Abbreviations are not provided for unannotated genes.

^a^ All DE results were determined with FDR <0.1.

## 4 Discussion

Studies have demonstrated that EHC has had positive effects on thermotolerance in chick embryos and broiler chicks (Piestun et al., 2013). Heat shock proteins (HSPs) were measured and found to increase simultaneously with thermotolerance development, suggesting that HSPs are essential for survival and provide protective mechanisms (Amaz et al, 2024a; 2024b). Although differences were also observed in the gut and adipose tissue, metabolism-related effects in the brain are unknown (Sulaiman et al., 2024; Beck et al., 2024). Previous studies have also shown that EHC increases the expression of anti-inflammatory genes in the hypothalamus (Rosenberg et al., 2020; Rosenberg et al., 2022). The objective of this study was to identify differences in the transcriptomes of the nCPa, POAH, and PVN resulting from embryonic heat conditioning and in response to a post-hatch heat challenge (which we anticipated would accentuate changes related to effects of EHC on thermotolerance). The heat challenge was introduced on day 4 post-hatch to align with our previous studies on appetite regulation and metabolism, which were also conducted on day 4 post-hatch to facilitate free-hand intracerebroventricular injections (Bohler et al., 2020; Sulaiman et al., 2024; Beck et al., 2024). Chicks were observed to be panting, a primary indication of heat expenditure and stress. Additionally, we hypothesized that EHC is associated with epigenetic changes, as previously demonstrated by Rosenberg et al. (2020). As chick age increases, environmental factors have the potential to “mute” epigenetic programming that occurs during embryonic development. We hypothesize that these epigenetic changes will lead to persistent effects on gene expression and transcriptional regulation. Accordingly, our design included hour 0 as a baseline to capture the effects of the EHC on gene expression and the heat stress timepoints to measure the effects of EHC on transcriptional regulatory effects that are not manifested without an external stressor.

RNA-seq analysis suggests that EHC influenced gene expression, with a total of 491 DEGs across tissues, with most localized in the nCPa. Almost no DEGs were detected in the PVN, in which we hypothesized that stress-related factors might be affected. In all tissues, no DEGs were identified at hour 0, suggesting that in the absence of a stressor, gene expression profiles were similar in control and EHC chicks. At hour 2 of the heat challenge, a few DEGs were detected in all three tissues, suggesting that although the heat exposure revealed differences in transcriptional regulation that were not apparent in the absence of a stressor, relatively few genes were affected (4 in PVN, 18 in POAH, and 2 in the nCPa). Although there were no DEGs detected in the PVN or POAH at hour 12, 467 DEGs were identified in the nCPa, suggesting that this region was more susceptible to the effects of heat challenge on transcriptional regulation and that the 2 h of heat exposure was not sufficient for transcriptional changes to be revealed. It was also interesting to note that the few DEGs detected in the POAH and PVN at hour 2 were more highly expressed in the EHC group than in the control group, whereas in the nCPa, most DEGs identified at hour 2 and those highlighted by the enrichment analysis at hour 12 were downregulated by EHC. This suggests that the associated pathways might have reduced activity during heat stress in chicks that were subjected to EHC. Additionally, the nCPa was the only nucleus in which effects were observed with time during the heat stress, with most occurring at 12 h. This implies that this region is more responsive to the effects of acute heat stress than the POAH or PVN.

All differentially expressed genes in the EHC group relative to the control group were individually investigated, exception for those identified at hour 12 in the nCPa, where only the top 10 upregulated and 10 downregulated genes were further analyzed and discussed below. At hour 2 in the nCPa, two genes were differentially expressed. Keratin 13 (KRT13) is involved in the maintenance of mucosal stratified squamous epithelial cells (Kalabusheva et al., 2023), and phosphoserine aminotransferase 1 (PSAT1) is related to high feed efficiency in Iranian native turkeys (Pezeshkian et al, 2022a; 2022b). However, both genes were downregulated, suggesting a decreased level of expression in EHC. If these pathways are less active in EHC chicks, it might indicate an energy-saving mechanism that enhances their efficiency in coping with the energetic demands of heat dissipation during heat stress.

### 4.1 Identification and physiological relevance of DEGs in the nCPa

Of the top 10 upregulated and 10 downregulated DEGs at hour 12 in the nCPa, two genes had a correlation with muscle and body weight. Secretogranin V (SCG5) expression in congenic mice was correlated with decreased body weight (Farber et al., 2008). SCG5 was downregulated in our RNA-seq analysis. Body weight was measured, and there were no significant differences between groups; however, this downregulation could suggest that EHC chicks may have sustained body weight through the entire growth period more than the control group when presented with heat stress. This could propose future studies where EHC chicks are kept through maturation and heat-challenged at a later period. Eukaryotic translation initiation factor 4A2 (EIF4A2) was also downregulated in EHC chicks. EIF4A2 is correlated with the formation and development of muscle tissue in swine; however, the underlying regulatory processes are still unknown (Wang et al., 2007). To our knowledge, no existing literature reports SCG5 or EIF4A2 being investigated in chick models. There were also genes identified related to inhibiting apoptosis, metabolic genes, and immune response. Apoptosis-resistant E3 ubiquitin protein ligase 1 (AREL1) inhibits apoptosis (Jo et al., 2021), which may prolong cell life in EHC chicks. Upregulated genes related to metabolism include ATP synthase F0 subunit 8 (ATP8), NADH dehydrogenase subunit 2 (ND2), and adenylate cyclase 9 (ADCY9). ATP8 encodes mitochondrial gene expression, which is important for providing energy to be used by the cell. In a study analyzing phytase supplementation and its effects on broiler growth, meat quality, and muscle myopathies such as woody breast syndrome, ATP8 was upregulated, indicating abundant intracellular ATP (Walk et al., 2024). For EHC chicks, ATP8 was downregulated, which may suggest less ATP synthesis and energy conservation at the cellular level. ND2 was also downregulated in EHC chicks. ND2 is a component of the NADH dehydrogenase complex, which is responsible for catalyzing NADH to ubiquinone and facilitating proton pumping out of the mitochondrial matrix (Čermáková et al., 2021). In a normal homeostatic state, the downregulation of ND2 would be considered a problem due to lack of catalyzation; however, downregulated ND2 in EHC chicks may imply energy-conserving properties. Genes related to inflammation and immune response include TBC1 domain family member 20 (TBC1D20), exportin 6 (XPO6), and proteasome subunit alpha 2 (PSMA2). TBC1D20 expression mediates autophagy in mice and was upregulated in EHC chicks (Sidjanin et al., 2016). With autophagy being important for removing damaged proteins and organelles at the cellular level, it can be assumed that TBC1D20 maintains cellular health and homeostasis in EHC chicks. Upregulated XPO6 showed the activation of the NF-kB inflammatory response signaling pathway in pulmonary monocytes in mice (Wu et al., 2024) and was upregulated in EHC chicks. This could suggest cellular protection from the NF-kB inflammatory response being activated in EHC. PSMA2 was the only downregulated gene associated with inflammation and the immune response. In a study with PSMA2 knockdown, dysregulation of signaling pathways involving the immune system and signal transduction was widely affected (Rashid et al., 2023). With PSMA2 being downregulated in EHC, this means that pathways involving signaling transduction and cellular health are lower than those in the controls. This could suggest that the signaling pathways involving PSMA2 are being energetically conserved in EHC chicks; however, additional research is needed to confirm this hypothesis.

### 4.2 Identification and physiological relevance of DEGs in the POAH

The POAH exhibited one downregulated and 17 upregulated DEGs, all at hour 2 of the heat challenge, and none had any effect on appetite, thermotolerance, or stress regions. In addition, none of those nine genes had an associated gene name or description as they are not annotated in the genome. This suggests future avenues of research to understand the pathways and actions of these unidentified genes. Although the DEGs in this group did not appear to cluster into a specific function or pathway, some relevant roles are noted. The C1q and TNF-related 8 (C1QTNF8) is an inflammation-related factor, and immune cross-tolerance was shown to be a consequence of EHC (Rosenberg et al., 2020; 2021; 2022) Microfibril-associated protein 5 (MFAP5) is downregulated during adipogenesis in patients with femoral head necrosis (Zhang et al., 2023). Although this study was carried out in humans, the adipogenic properties of MFAP5 could be translatable to the chicks in our study. MFAP5 was upregulated in the EHC group, and this could suggest adipocyte formation is reduced in EHC chicks compared to controls, potentially indicating thermoregulatory mechanisms mediated by the POAH. Serpin family F member 1 (SERPINF1) was also upregulated in EHC. SERPINF1 was found to be anti-angiogenic and prevents new blood vessels from forming in triple-negative breast cancer (Maiti et al., 2019). Relevance to EHC could be speculated by promoting centralized thermoregulation through decreasing new blood vessel formation in the body of the EHC chicks. The only downregulated gene, BEN domain containing 3 (BEND3), has not been shown, to our knowledge, to have direct biological functions. However, it is essential in transcriptional repression and actively binds with heterochromatin in the nucleus, making it an essential binding protein (Shiheido and Shimizu, 2015). Overall, the genes noted are indicative of EHC potentially improving thermotolerance, which aligns with the mechanistic action of the POAH.

### 4.3 Identification and physiological relevance of DEGs in the PVN

The PVN produced four DEGs specifically at hour 2. Two of them stand out as factors that may play a role in hypothalamic regulation of the water and salt balance. They are vasoactive intestinal peptide receptor 1 (VIPR1) and caprin family member 2 (CAPRIN2), both of which were upregulated in EHC chicks. Signaling through VIPR1 has previously been demonstrated to decrease food intake in mice, and Yu et al. (2008) showed that mice treated with a VPAC1 (alias of VIPR1) agonist also showed inhibited food intake. In addition, VPAC1 also delayed development and reduced weight in mice (Fabricius et al., 2011). Although food intake was not measured in our study, the effects of decreased food intake in mice could be translated to EHC chicks being more energetically efficient during heat stress and maintaining their body weight during this stress, thus providing more energy to protect and maintain homeostasis in their visceral organs. The endogenous ligand for VPAC1, vasoactive intestinal peptide, is involved in smooth muscle relaxation, hormone release, and water and mineral movement in the gut. CAPRIN2 is vastly increased in the nucleus and cytoplasm of arginine vasopressin (AVP) neurons during dehydration in rats (Bárez-López et al., 2022) and may affect thermotolerance due to its water-regulating properties. It is tempting to speculate that *via* the PVN, the expression of these factors may regulate salt and water retention during heat stress. In endotherms, body temperature and water balance are strongly associated, and in chickens, evaporative heat loss can be facilitated through increased panting during heat stress (also making them susceptible to alkalosis).

### 4.4 Identification and physiological relevance of DEGs with timepoint interactions

Although there were only DEGs at timepoint interactions at hour 12, these observed time effects were also of importance. We chose to analyze timepoint interactions to compare the different hours of heat exposure on the tissues themselves, without treatment being compared. While we anticipated more DEGs across tissues other than the nCPa, these data provided insights on gene expression as the heat challenge progressed. Hour 12 vs 2 yielded nine genes, and upon further investigation, two were of importance. Ubiquitin-associated protein 2 like (UBAP2L), when triggered by stress, plays a role in processing body (PB) formation and PB interaction with stress granules (Riggs et al., 2024). Because UBAP2L was upregulated at hour 12 relative to that at hour 2, this suggests that a prolonged stress cascade from the heat challenge increased the expression. BCL2 family apoptosis regulator (BCL1) is anti-apoptotic, and the BCL2 family plays a role in inhibiting mitophagy, which are both essential in regulating reactive oxygen species (ROS) (Su et al., 2023). ROS production can be stimulated during heat stress by causing oxidative stress, so it is plausible that because BCL1 was downregulated, heat stress at hour 12 vs. 2 caused increased production of ROS throughout the heat challenge.

Moving into hour 12 vs. hour 0, there were 49 DEGs between these timepoints. With a greater number of genes becoming activated as time progressed, the heat challenge can be noted as a dynamic process. This result is expected as hour 0 was set as the baseline for the heat challenge and hour 2 and 12 were collected afterward to measure any resulting differences. Of the 49 genes, 13 had no title or name association, showing that they are not annotated in the chicken genome. The remaining genes were investigated, and genes discussed below were associated with cellular metabolism and/or stress regulation and had relevance to changes associated with heat.

Metabolic genes include mannosyl-oligosaccharide glucosidase (MOGS), metadherin (MTDH), UDP-galactose-4-epimerase (GALE), twinkle mtDNA helicase (C10orf2), ATP synthase F0 subunit 8 (ATP8) and subunit 6 (ATP6), suppressor of glucose, autophagy-associated 1 (SOGA1), NADH dehydrogenase subunit 5 (ND5), subunit 3 (ND3), and subunit 4 (ND4), CCR4-NOT transcription subunit 4 (CNOT4), and cytochrome B. Each of these genes had regulatory effects in most phases of cellular metabolism. In particular, genes related to ATP and NADH regulation were present, where ATP8 was upregulated and increased intracellular ATP abundance (Walk et al., 2024), and ATP6 was also upregulated, which has been shown to facilitate the flow of protons across the mitochondrial membrane and drive ATP synthesis for energy production. ATP6 was shown to be decreased when lipopolysaccharide-induced intestinal oxidative stress is present. This suggests that chicks at 12 h had reduced oxidative stress, thus upregulating the gene. ND3, ND4, and ND5 were also all upregulated, and all of these are involved in the mitochondrial electron transport chain. This implies that at hour 12, cellular function was not disrupted at the mitochondrial level. In addition, C10orf2 has been shown to cause mitochondrial DNA depletion syndrome in infants (Remtulla et al., 2019) and was found to be downregulated in chicks at hour 12. To further support the idea of mitochondrial preservation, the CNOT4 complex, which regulates RNA metabolism (Collart, 2016), was also upregulated at hour 12. CYTB was upregulated at hour 12 and affects energy metabolism through oxidative phosphorylation. In a study carried out on sheep, CYTB increased thermodynamic stability and energy metabolism (Pal et al., 2019). Moreover, some cellular metabolism genes were also downregulated, such as MOGS and MTDH. MTDH regulates vascular endothelial growth factor expression *via* the PI3/AKT pathway, and the PI3/AKT pathway is also responsible for cell metabolism and growth (Zhu et al., 2015). MOGS was also a surprising gene to be downregulated, given its important role in cellular metabolism. A previous study showed that the knockdown of MOGS led to increased proliferation and differentiation of Schwann cells (Zhang et al., 2022). Schwann cells play an active role in forming the myelin sheath, which covers the axon and facilitates faster nerve signal transmission (Zhang et al., 2022). GALE at 12 h was downregulated, so processes were muted. The overexpression of GALE increases gluconeogenesis, and it is an enzyme that converts galactose to glucose (Zhu et al., 2017). The downregulation of these genes could be because of a variety of factors; however, at hour 12, genes had prolonged expression, causing them to decrease over time. SOGA1 was also downregulated at hour 12. However, SOGA1 promotes AMPK, which further upregulates PDK4, which is a protein that decreases glucose metabolism. Therefore, SOGA1 being downregulated at hour 12 could promote glucose metabolism (Wei et al., 2023).

There were two stress-associated genes that were also found in 12 vs 0, which are the gem nuclear organelle associated protein 4 (GEMIN4) and G3BP stress granule assembly factor 1(G3BP1). Both these genes were downregulated at hour 12 and have important effects on stress responses. G3BP1 is an RNA binding protein associated with the assembly of stress granules following a stimulus (Martin et al., 2013). Downregulation could be due to an expression plateau, where G3BP1 had higher expression at time 0, and as the heat challenge progressed, there was a decline in expression. Another noteworthy gene was GEMIN4, which functions as a novel co-regulator of the mineralocorticoid receptor (MR) and actually decreases MR activity (Yang et al., 2015). MRs are responsible for mediating the action of aldosterone and cortisol, which are widely changed due to stress.

With timepoint interactions being irrespective of treatment, it is worth speculating whether or not EHC had a strong influence on these DEGs. Regardless, most of the genes described were of benefit to the physiological and molecular health of the chicks. Given that 12 h is a substantial amount of time, we expected to observe many genes downregulated and/or a cessation of metabolic processes to come to a halt because of the prolonged stress that heat imposes on the body. However, these data show that cellular resiliency is much more of a theme than anticipated.

The punch biopsy isolation of individual nuclei afforded superior anatomical resolution in parsing out effects of gene expression that were region-specific. These regions were selected based on previous work (Kuenzel and van Tienhoven, 1982; Bohler et al., 2020). CRH is a major neuron projected onto the PVN from the nCPa for mediating the stressor response; however, our results did not contain any notable levels of CRF being expressed in the nCPa or PVN. Other genes that were expected to be differentially expressed were HSPs, adrenocorticotropic hormone (ACTH), a stress modulator, appetite-regulating factors such as NPY, and melanocortin ligands such as POMC and a-MSH. Within our RT-PCR data, which are provided as supplementary tables, NPY, CRF, POMC, CCK, UCN3, TRH, and HSP90 mRNA abundance was measured in each of the nuclei. RT-PCR was measured before RNA-seq genome annotation. In the PVN, CRF was significant between control and EHC. In addition, there was significance in timepoint differences for NPY, POMC, UCN3, and TRH. HSP90 also displayed significant differences for timepoint–treatment interactions. For the POAH, CRF was also significant between control and EHC; POMC was significant for treatment, timepoint, and treatment–timepoint interactions, and HSP90 was significant between treatments. It is especially relevant that the RT-PCR data for the nCPa had the least amount of differences—with only NPY showing statistical significance between timepoints. Considering that the nCPa was the highest-yielding tissue for DEGs in the RNA-seq analysis, this difference in expression from RNA-seq to RT-PCR was intriguing. It is also important to note that these were not identified as differentially expressed. Additionally, genes tested with RT-PCR could not be found in any of the RNA-seq raw data. Reasons for this could be because of the large number of genes in the genome and that RT-PCR is a targeted assay.

Previous studies have shown that chicks on day 4 have differences in stress- and appetite-related gene expression in both the PVN and nCPa, particularly in brain-derived neurotrophic factor (BDNF) and CRF. However, it is important to note that in the experiment performed by Bohler et al. (2020), chicks were intracerebroventricularly injected with either NPY, astressin, a-MSH—direct regulators of appetite—or CRH, which directly affects stress modulation (Bohler et al., 2020). Although feed intake was not measured in this study, it is also possible that decreased feed intake in both groups could have reduced the differential expression related to appetite-regulating genes. When chicks experience hyperthermia, production aspects such as growth and reproduction decrease to maintain organ homeostasis (Moraes et al., 2003; Wolfenson et al., 1981). Thus, heat exposure also decreases feed consumption to reduce thermogenic effects on metabolization and nutrient partitioning (Mckee et al., 1997). This may explain why no appetite-regulating genes were found to be differentially expressed during the heat challenge.

To conclude, EHC has been credited with reducing the severity of heat stress on hypothalamic responses, and further research is needed to determine whether the identified DEGs play a direct role in heat stress tolerance in chicks. Additionally, some of these studies were conducted in non-chick models and may involve mammalian models where respiration is different (active vs positive) from that in avians, which means that physiological and molecular responses may differ. Future research could explore whether the duration of heat exposure influences gene expression (e.g., longer exposure periods), whether age affects these responses, and whether reduced feed intake contributes to muted hypothalamic activity. Combined with previous literature on EHC, our transcriptomic analysis suggests that the gene expression profile of the hypothalamus and the different nuclei resembles patterns that align with remediated stress in EHC chicks. This study enhances our understanding of the molecular mechanisms underlying DEG expression in response to high ambient temperatures.

## Data Availability

The data presented in the study are deposited in the GEO repository, accession number GSE298648.
