# Supplementary material for: Embryonic thermal conditioning and post-hatch heat challenge alter hypothalamic expression of genes related to appetite, thermoregulation, and stress modulation in broiler chicks
Source: Front Physiol. 2025 Jun 17;16:1583958. doi: 10.3389/fphys.2025.1583958 (PMC12208832; doi:10.3389/fphys.2025.1583958)
Supplement: Supplementary file 1 [file Supplementaryfile1.docx]

**Supplementary Materials**


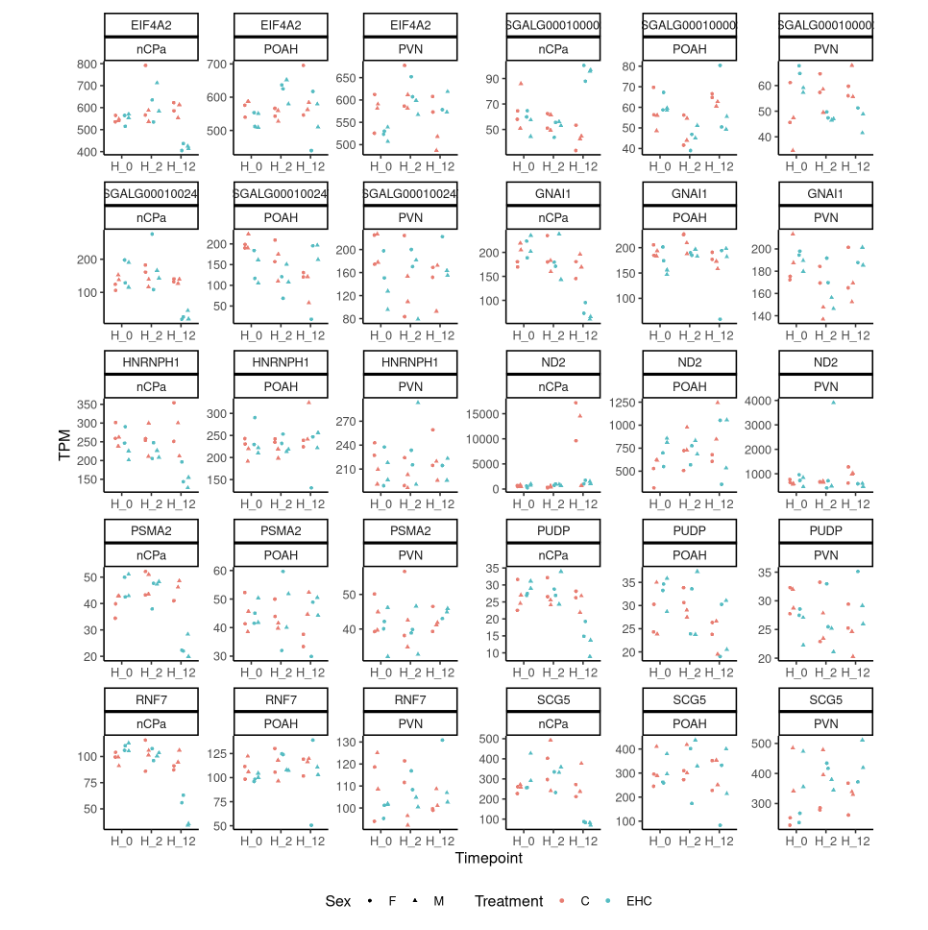


Supplementary Figure 1. Top 10 DEGs in hour 12 nCPa EHC tissue. FDR > 0.1, does not account for upregulated/downregulated DEGs.


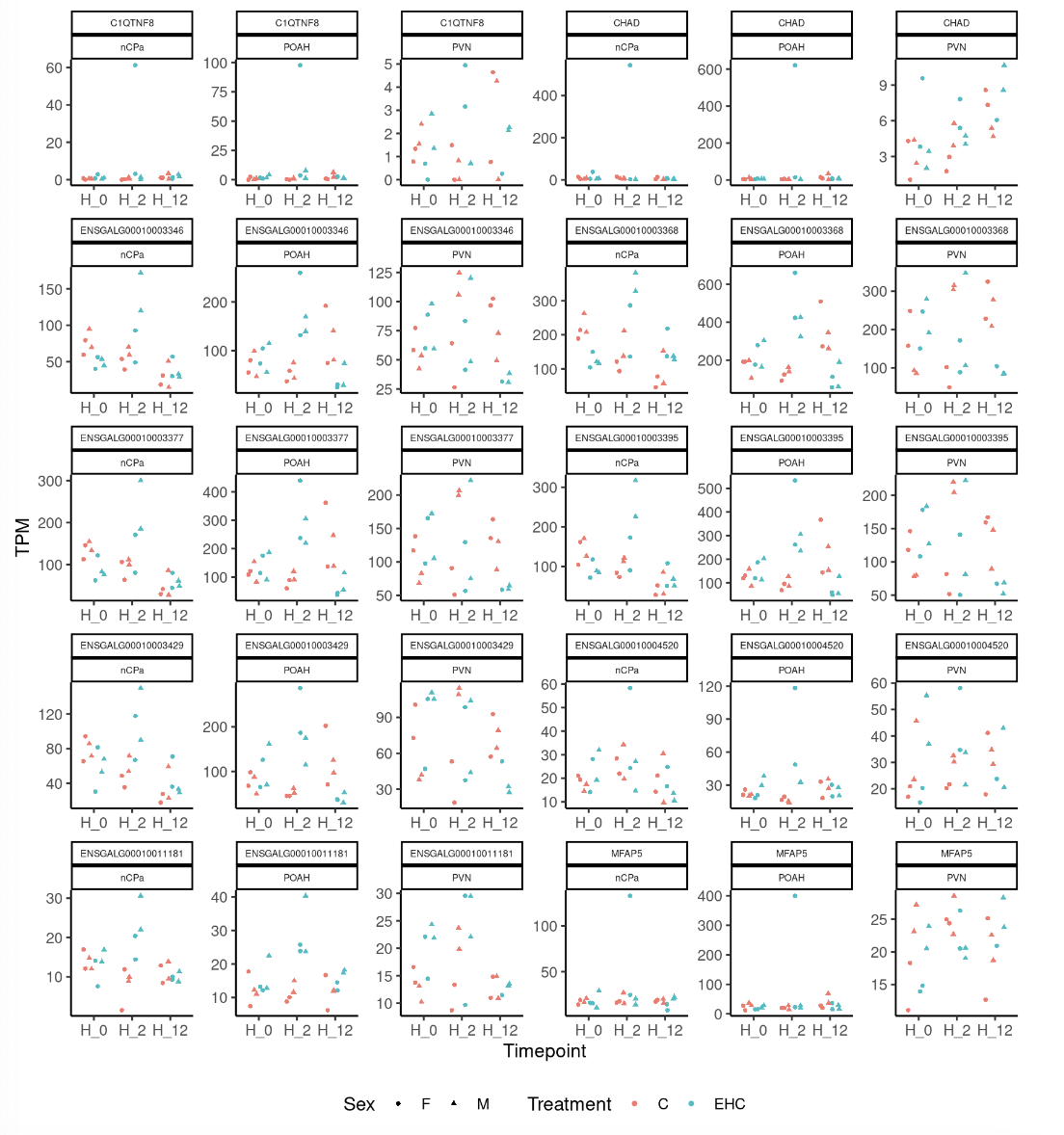


Supplementary Figure 2. Top 10 DEGs in hour 2 POAH EHC tissue. FDR > 0.1, Outliers were not eliminated, note- C1QTNF8, CHAD, MFAP5. Based strictly on FDR value, Upregulated and Downregulated Genes were not accounted for.

Supplementary Table 1: Summary of relative transcript abundance and statistical analysis in the paraventricular nucleus^1^.

| Effect | *NPY* | *CRF* | *POMC* | *CCK* | *UCN3* | *TRH* | *HSP90* |
| --- | --- | --- | --- | --- | --- | --- | --- |
| Treatment |  |  |  |  |  |  |  |
| Control | 2.44 ± 0.92 | 3.19 ± 0.54 | 0.99 ± 0.16 | 0.75 ± 0.14 | 1.83 ± 0.42 | 1.47 ± 0.28 | 1.16 ± 0.18 |
| EHC | 3.52 ± 0.85 | 1.54 ± 0.52 | 1.27 ± 0.17 | 0.79 ± 0.13 | 1.15 ± 0.39 | 1.17 ± 0.29 | 1.33 ± 0.17 |
| *p*-value | 0.3915 | **0.0334** | 0.2556 | 0.8126 | 0.2409 | 0.4605 | 0.4795 |
| Timepoint |  |  |  |  |  |  |  |
| 0 | 1.68 ± 1.06 | 1.78 ± 0.61 | 1.68 ± 0.21^A^ | 1.07 ± 0.16 | 2.87 ± 0.49^A^ | 1.72 ± 0.34^A^ | 1.52 ± 0.21 |
| 2 | 5.29 ± 1.11 | 3.11 ± 0.67 | 0.79 ± 0.19^B^ | 0.73 ± 0.17 | 0.44 ± 0.49^B^ | 0.45 ± 0.37^B^ | 1.05 ± 0.21 |
| 12 | 1.97 ± 1.07 | 2.21 ± 0.67 | 0.92 ± 0.21^B^ | 0.52 ± 0.17 | 1.17 ± 0.51^AB^ | 1.79 ± 0.37^A^ | 1.16 ± 0.22 |
| *p*-value | **0.0447** | 0.3431 | **0.0087** | **0.0784** | **0.0038** | **0.0176** | **0.2701** |
| Timepoint X Treatment | 0.5236 | 0.4621 | 0.1181 | 0.8352 | 0.3664 | 0.4966 | **0.0459** |

^1^ All results are expressed as least square mean ± standard error. For this experiment, there were 20 chicks per timepoint. Letters indicate p < 0.05, post-hoc tukey test. Abbreviations: Neuropeptide Y (NPY), corticotropin-releasing factor (CRF), proopiomelanocortin (POMC), Leptin (LEP), cholecystokinin (CCK), urocortin-3 (UCN3), thyrotropin-releasing hormone (TRH), Heat Shock Protein 90 (HSP90).

Supplementary Table 2: Summary of relative transcript abundance and statistical analysis in the pre-optic anterior-hypothalamus^1^.

| Effect | *NPY* | *CRF* | *POMC* | *CCK* | *UCN3* | *TRH* | *HSP90* |
| --- | --- | --- | --- | --- | --- | --- | --- |
| Treatment |  |  |  |  |  |  |  |
| Control | 4.32 ± 2.01 | 0.94 ± 0.52 | 3.71 ± 0.60 | 1.95 ± 1.35 | 0.91 ± 0.31 | 1.46 ± 0.36 | 0.88 ± 0.17 |
| EHC | 4.18 ± 1.68 | 2.47 ± 0.46 | 1.78 ± 0.52 | 4.09 ± 1.14 | 0.88 ± 0.28 | 1.14 ± 0.35 | 1.56 ± 0.15 |
| *p*-value | 0.9572 | **0.0335** | **0.0205** | 0.2336 | 0.9460 | 0.5202 | **0.0054** |
| Timepoint |  |  |  |  |  |  |  |
| 0 | 1.54 ± 2.17 | 1.67 ± 0.59 | 2.00 ± 0.64^B^ | 5.59 ± 1.48 | 0.89 ± 0.35 | 1.10 ± 0.44 | 1.59 ± 0.19 |
| 2 | 8.64 ± 2.17 | 1.71 ± 0.56 | 1.29 ± 0.59^B^ | 2.26 ± 1.43 | 0.77 ± 0.35 | 1.14 ± 0.41 | 0.96 ± 0.19 |
| 12 | 2.56 ± 2.52 | 1.73 ± 0.64 | 4.94 ± 0.82^A^ | 1.21 ± 1.66 | 1.03 ± 0.39 | 1.65 ± 0.46 | 1.10 ± 0.22 |
| *p*-value | 0.0569 | 0.9970 | **0.0033** | 0.1217 | 0.8886 | 0.6335 | 0.0647 |
| Timepoint X Treatment | 0.9429 | 0.6178 | 0.0006 | 0.0997 | 0.7998 | 0.1255 | 0.1810 |

^1^ All results are expressed as least square mean ± standard error. For this experiment, there were 20 chicks per timepoint. Letters indicate p < 0.05, post-hoc tukey test. Abbreviations: Neuropeptide Y (NPY), corticotropin-releasing factor (CRF), proopiomelanocortin (POMC), Leptin (LEP), cholecystokinin (CCK), urocortin-3 (UCN3), thyrotropin-releasing hormone (TRH), Heat Shock Protein 90 (HSP90).

Supplementary Table 3: Summary of relative transcript abundance and statistical analysis in the nucleus of the hippocampal commissure^1^.

| Effect | *NPY* | *CRF* | *POMC* | *CCK* | *UCN3* | *TRH* | *HSP90* |
| --- | --- | --- | --- | --- | --- | --- | --- |
| Treatment |  |  |  |  |  |  |  |
| Control | 3.91 ± 1.23 | 2.20 ± 0.76 | 0.82 ± 0.19 | 1.86 ± 0.44 | 0.26 ± 0.33 | 0.43 ± 0.54 | 0.88 ± 0.19 |
| EHC | 2.75 ± 1.11 | 2.52 ± 0.63 | 0.79 ± 0.14 | 1.13 ± 0.37 | 1.03 ± 0.29 | 1.01 ± 0.45 | 1.25 ± 0.18 |
| *p*-value | 0.4912 | 0.7512 | 0.8913 | 0.2153 | 0.0849 | 0.4162 | 0.1607 |
| Timepoint |  |  |  |  |  |  |  |
| 0 | 1.28 ± 1.31^B^ | 3.83 ± 0.74 | 0.65 ± 0.19 | 0.69 ± 0.45 | 0.97 ± 0.34 | 0.90 ± 0.55 | 1.28 ± 0.22 |
| 2 | 7.55 ± 1.59^A^ | 1.53 ± 0.82 | 1.11 ± 0.22 | 1.74 ± 0.49 | 0.41 ± 0.41 | 0.49 ± 0.64 | 1.02 ± 0.25 |
| 12 | 1.16 ± 1.38^B^ | 1.72 ± 0.98 | 0.65 ± 0.22 | 2.06 ± 0.55 | 0.55 ± 0.38 | 0.77 ± 0.63 | 0.90 ± 0.22 |
| *p*-value | **0.0054** | 0.0920 | 0.2257 | 0.1290 | 0.5292 | 0.8872 | 0.4774 |
| Timepoint X Treatment | 0.2655 | 0.6682 | 0.7275 | 0.4367 | 0.2819 | 0.5730 | 0.5130 |

^1^ All results are expressed as least square mean ± standard error. For this experiment, there were 20 chicks per timepoint. Letters indicate p < 0.05, post-hoc tukey test. Abbreviations: Neuropeptide Y (NPY), corticotropin-releasing factor (CRF), proopiomelanocortin (POMC), Leptin (LEP), cholecystokinin (CCK), urocortin-3 (UCN3), thyrotropin-releasing hormone (TRH), Heat Shock Protein 90 (HSP90).
